# Supplementary material for: Comparison of Structural and Short Variants Detected by Linked-Read and Whole-Exome Sequencing in Multiple Myeloma
Source: Cancers (Basel). 2021 Mar 10;13(6):1212. doi: 10.3390/cancers13061212 (PMC7999337; doi:10.3390/cancers13061212)
Supplement: Supplementary file 1 [file cancers-13-01212-s001.zip › Supplementary_Figures_Tables/Kumar_et_al_Supplementary_Figures.pdf]

Comparison of structural and short variants detected by linked-read and whole-exome sequencing in multiple myeloma

Ashwini Kumar[1-2], Sadiksha Adhikari[1-2], Matti Kankainen[2-5], Caroline A. Heckman[1-2]

1. Institute for Molecular Medicine Finland - FIMM, HiLIFE - Helsinki Institute of Life Science, iCAN Digital Cancer Medicine Flagship, University of Helsinki, Helsinki, Finland
2. iCAN Digital Precision Cancer Medicine
3. Medical and Clinical Genetics, University of Helsinki, Helsinki University Hospital, Helsinki, Finland
4. Translational Immunology Research Program and Department of Clinical Chemistry, University of Helsinki, Helsinki, Finland
5. Hematology Research Unit Helsinki, Department of Hematology, Helsinki University Hospital Comprehensive Cancer Center, Helsinki, Finland.

Supplementary figures and figure legends

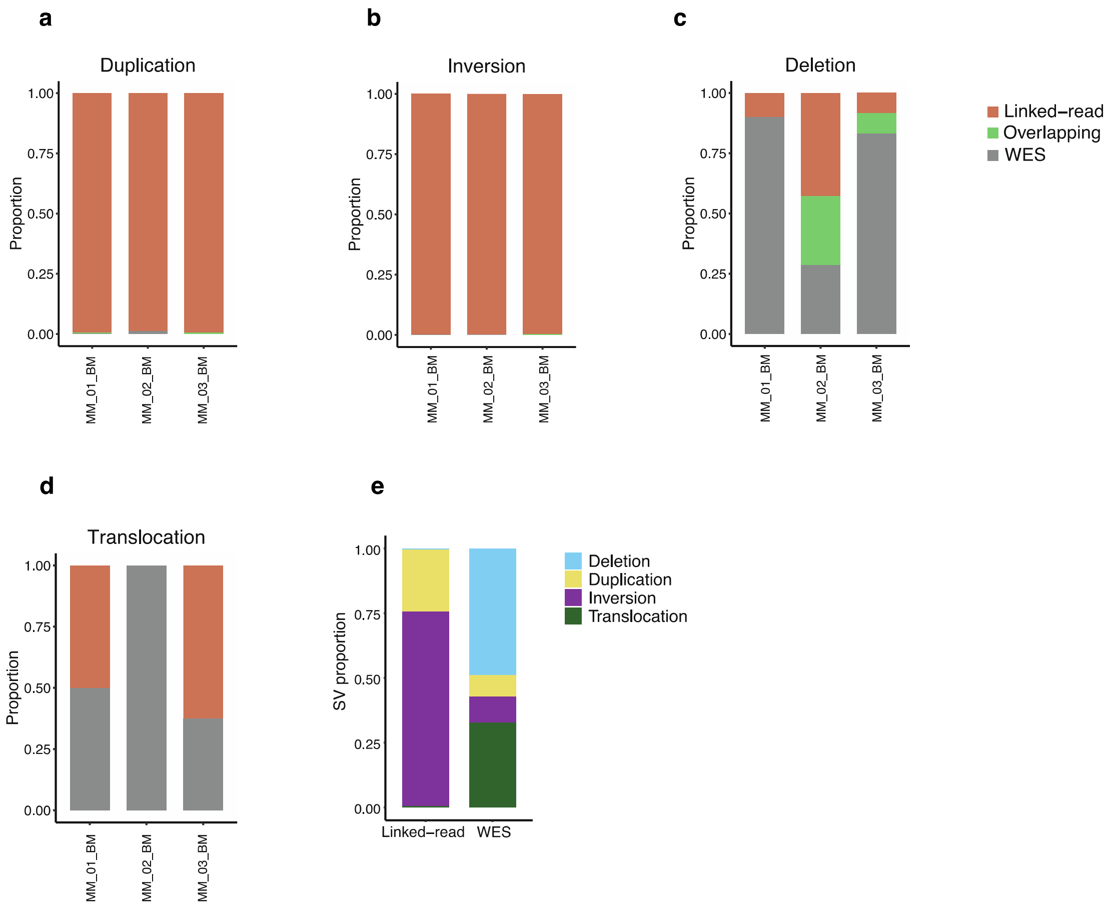

Supplementary Figure 1.

Total SVs detected by linked-read and WES in 3 paired samples.

(a) Duplication; linked-read sequencing detected more than 99% of all duplications across three samples (b) Inversion; linked-read detected more than 99% of inversions called in each sample. (c) Deletions; WES detected a higher proportion of deletions across all the samples. Linked-read sequencing alone detected about 10% of deletions in two samples and more than 40% in one sample. The proportion of overlaps varied across the samples. (d) Translocations; the proportion of translocations varied among the samples. WES detected all the translocations in one sample and 50% in another. (e) The proportion of each type of SV called by each sequencing method.

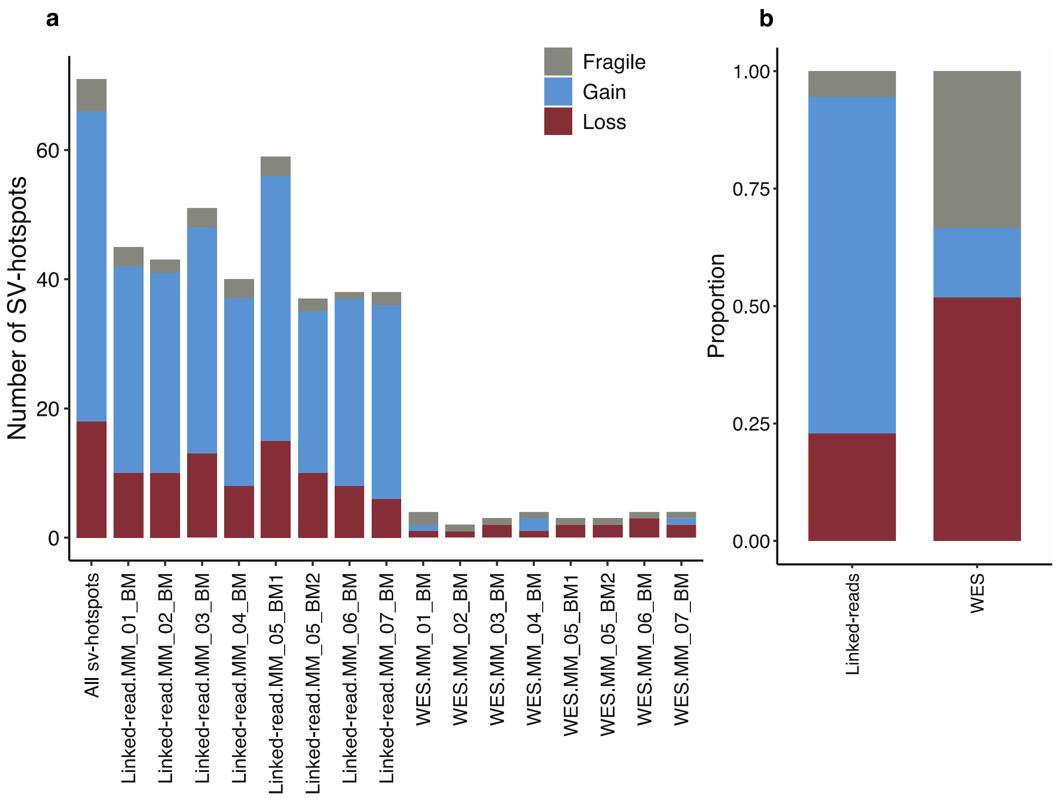

Supplementary Figure 2. The number of multiple myeloma specific SV hotspots across 8 samples identified by linked-read and WES. (a) The first bar shows the total number of identified SV hotspots. The median number of hotspots per patient is 2. The other bars represent hotspots identified in each sample by linked-read sequencing and WES. Linked-read sequencing detected an exceptionally high number of SVs per patient, with a median number of 41.5. The median number of SVs for WES is 3.5. (b) Overall linked-read sequencing detected more gain of function hotspots. WES detected a higher proportion of loss of function hotspots.

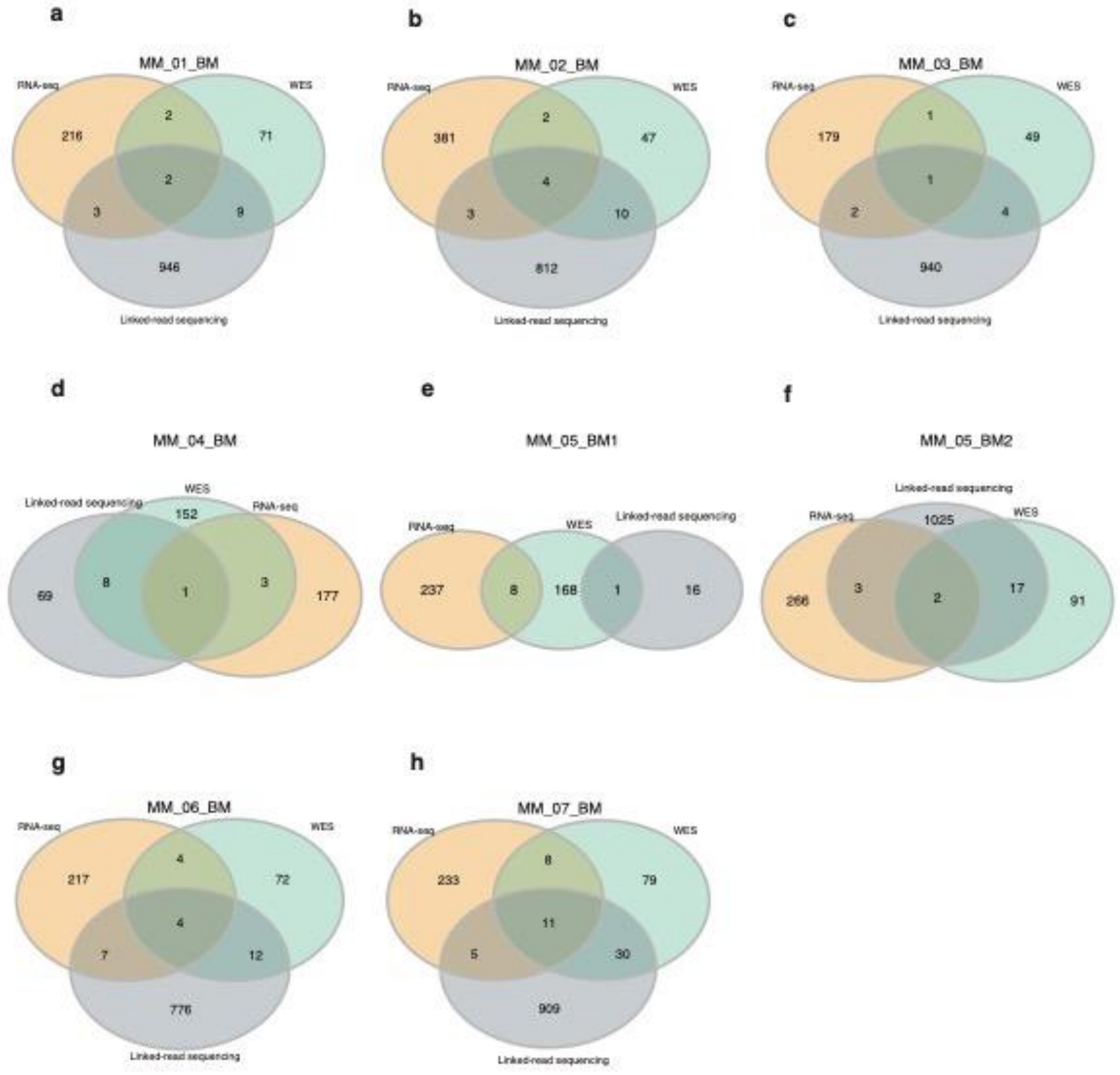

Supplementary Figure 3. The overlapping short variants across all 8 tumor samples identified by GATK tool RNA-seq, WES and linked-read exome sequence data.

a

KRAS, MM_04_BM

Linked-read

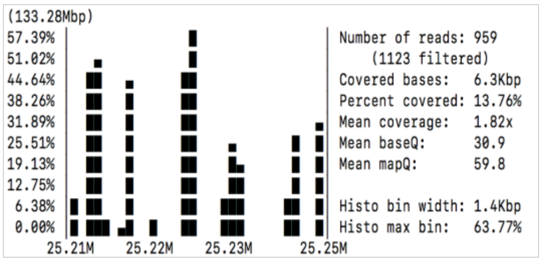

WES

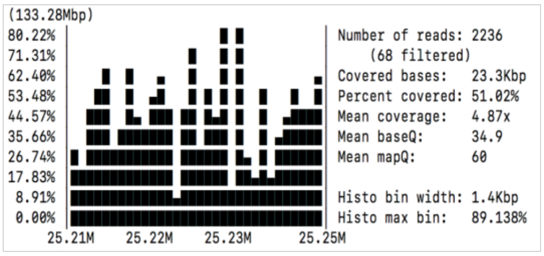

b

FAM46C; MM_05_BM1

Linked-read

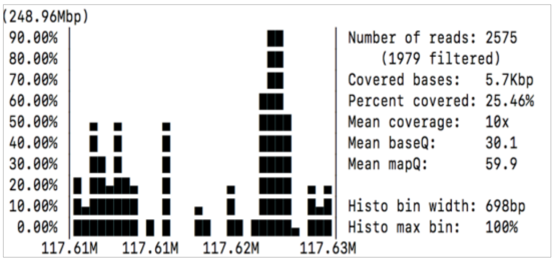

WES

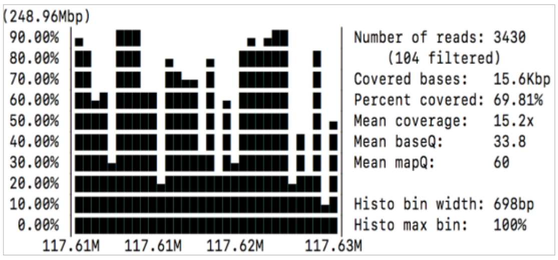

c

FAM46C; MM_07_BM

Linked-read

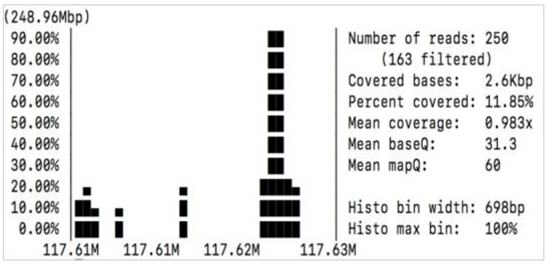

WES

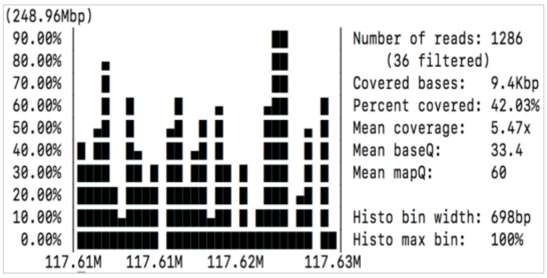

d

BRAF; MM_03_BM

Linked-read

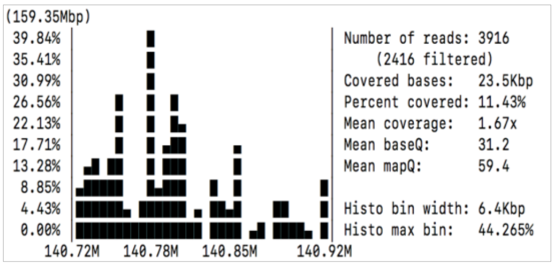

WES

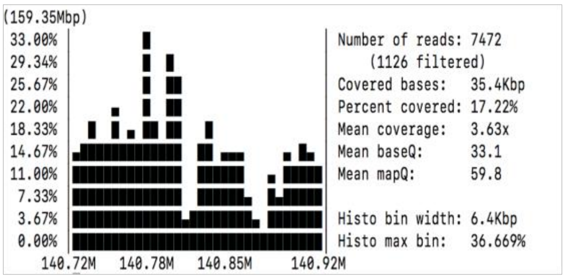

e

KRAS; MM_01_BM

Linked-read

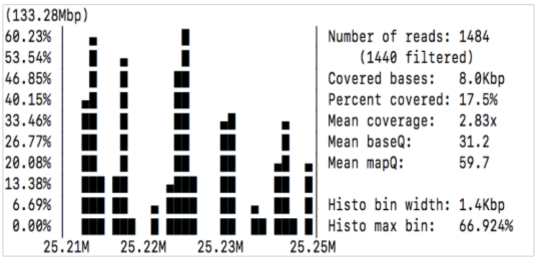

WES

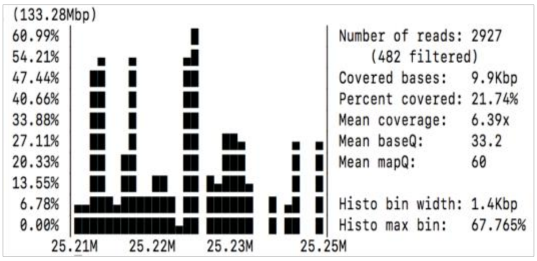

f

TP53; MM_01_BM

Linked-read

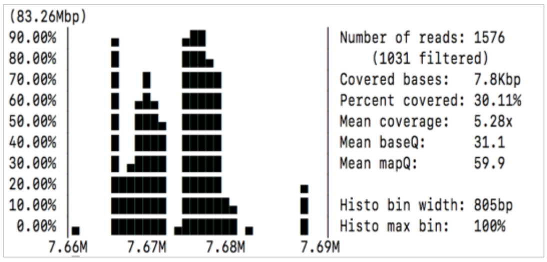

WES

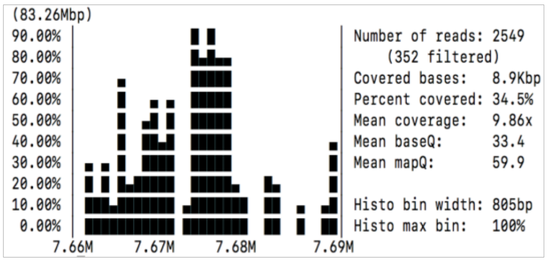

g

NRAS; MM_02_BM

Linked-read

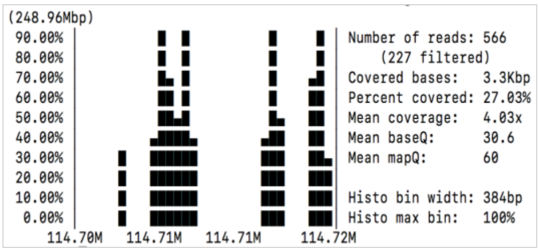

WES

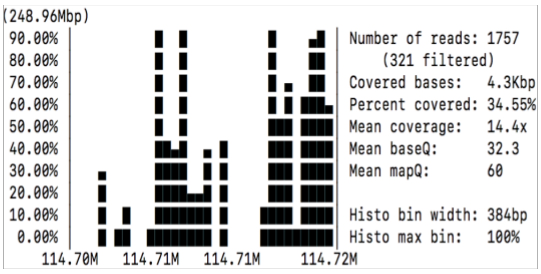

h

FAM46C, MM_05_BM2

Linked-read

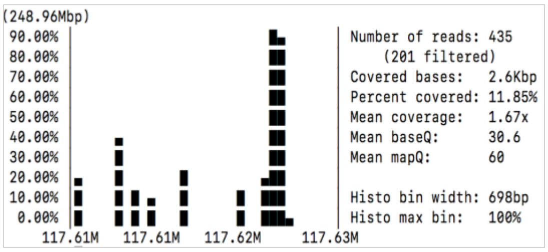

WES

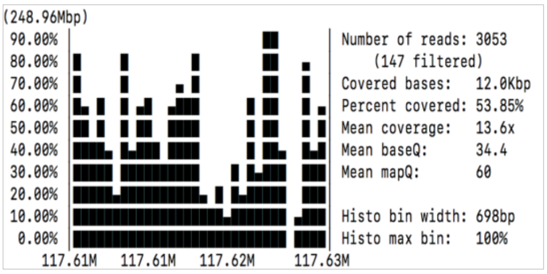

i

NRAS; MM_05_BM2

Linked-read

WES

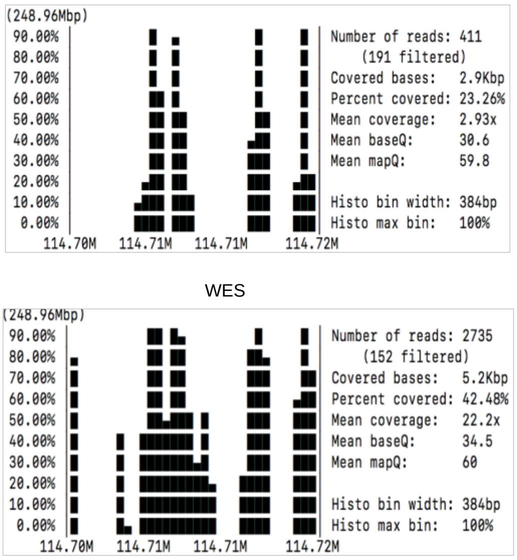

j

KRAS; MM_07_BM

Linked-read

WES

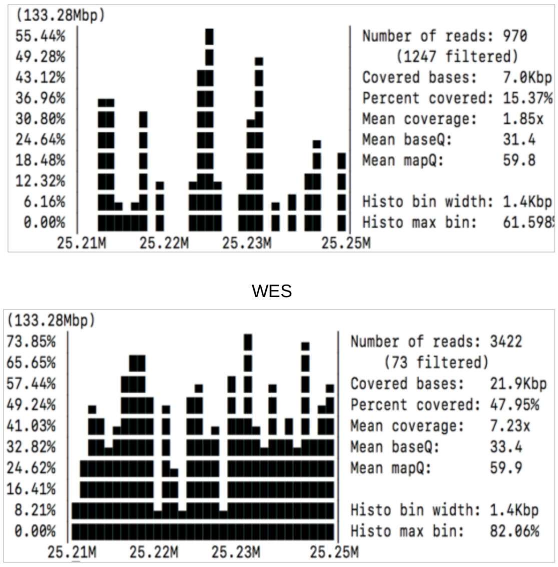

k

TP53; MM_07_BM

Linked-read

WES

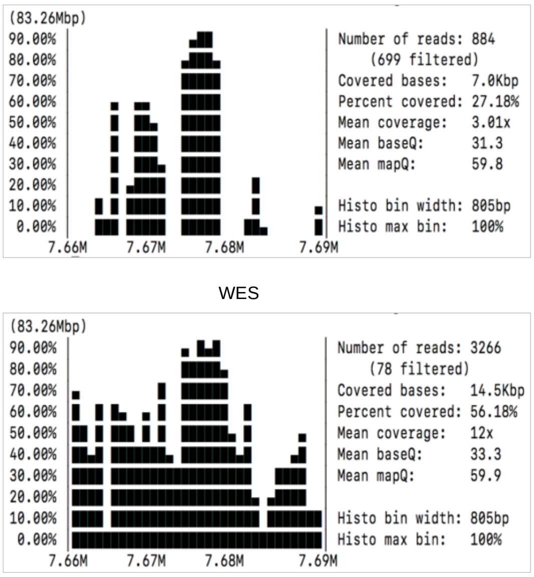

Supplementary Figure 4. Gene coverage plots depicting mutations in myeloma-specific genes in linked-read and WES data.
